# Enhancing Pharmacokinetics and Pharmacodynamics of Rosuvastatin Calcium through the Development and Optimization of Fast-Dissolving Films

**DOI:** 10.3390/pharmaceutics15112640

**Published:** 2023-11-19

**Authors:** Ibrahim Ashraf, Pierre A. Hanna, Shadeed Gad, Fathy I. Abd-Allah, Khalid M. El-Say

**Affiliations:** 1 Department of Pharmaceutics, Faculty of Pharmacy, Suez Canal University, Ismailia 41522, Egypt; dribrahim172@gmail.com (I.A.); pierre_hanna@pharm.suez.edu.eg (P.A.H.); shaded_abdelrahman@pharm.suez.edu.eg (S.G.); 2 Department of Pharmaceutics and Industrial Pharmacy, Faculty of Pharmacy, Al-Azhar University, Cairo 11651, Egypt; fathyfet@yahoo.com; 3 Department of Pharmaceutics, Faculty of Pharmacy, King Abdulaziz University, Jeddah 21589, Saudi Arabia

**Keywords:** fast-dissolving film, rosuvastatin, pharmacokinetics, hyperlipidemia, design of experiment approach

## Abstract

Rosuvastatin (RSV) is a widely used cholesterol-lowering medication, but its limited bioavailability due to its susceptibility to stomach pH and extensive first-pass metabolism poses a significant challenge. A fast-dissolving film (FDF) formulation of RSV was developed, characterized, and compared to the conventional marketed tablet to address this issue. The formulation process involved optimizing the thickness, disintegration time, and folding durability. All formulations were assessed for in vitro disintegration, thickness, folding endurance, in vitro dissolution, weight, and content uniformity. The study’s results revealed that the optimized RSV-FDF displayed a significantly faster time to maximum plasma concentration (t_max_) of 2 h, compared to 4 h for the marketed tablet. The maximum plasma concentration (C_max_) for the RSV-FDF (1.540 µg/mL ± 0.044) was notably higher than that of the marketed tablet (0.940 µg/mL ± 0.017). Additionally, the pharmacodynamic assessment in male Wistar rats demonstrated that the optimized RSV-FDF exhibited an improved lipid profile, including reduced levels of low-density lipoproteins (LDLs), elevated high-density lipoproteins (HDLs), decreased triglycerides (TGs), and lower very-low-density lipoproteins (VLDLs) compared to the conventional tablet. These findings underscore the potential of RSV-FDFs as a promising alternative to enhance the bioavailability and therapeutic efficacy of rosuvastatin in treating dyslipidemia. The faster onset of action and improved lipid-lowering effects make RSV-FDFs an attractive option for patients requiring efficient cholesterol management.

## 1. Introduction

Hyperlipidemia is defined as elevated levels of triglycerides and/or any of the following lipoproteins: very-low-density lipoproteins (VLDLs), low-density lipoproteins (LDLs), or high-density lipoproteins (HDLs). Hyperlipidemia expression is replaced by dyslipidemia as increasing HDL levels is a good sign [1]. Dyslipidemia is classified into familial (primary) dyslipidemia, which is caused by genetic disorders, and acquired (secondary) dyslipidemia, caused by the progression or signs of some diseases like diabetes, kidney disorder, and hypothyroidism [2,3]. Also, hyperlipidemia can increase the risk of developing some medical conditions like bladder cancer and coronary artery diseases [4]. Some cases report that dyslipidemia appears in overweight pediatrics. As a risk factor for management, triglycerides, total cholesterol, very-low-density lipoproteins (VLDLs), low-density lipoproteins (LDLs), and high-density lipoproteins (HDLs) are periodically analyzed for patients to prevent atherosclerosis [3]. The first treatment for controlling dyslipidemia is lifestyle management, e.g., decreasing fat and high-cholesterol diet intake. Several drug categories are used to manage the level of serum lipid. The first group is bile acid binders such as cholestyramine, colesovelam, and colestipol. The second group is fibrates, e.g., fenofibrate and gemfibrozil, which stimulate the cells’ fatty acid uptake, convert it to acyl-CoA derivatives, and then catabolize it via oxidative pathways [5]. The third lipid-lowering group is cholesterol absorption inhibitors, e.g., ezetimibe, which significantly decreases the absorbed quantity of cholesterol. The fourth group is considered a supplement rather than a drug, which is omega-3 fatty acids that act by inhibiting VLDL synthesis. The fifth and most common group used for managing dyslipidemia is the 3-hydroxy-3-methyl-glutaryl-coenzyme A (HMG-COA) reductase inhibitors (statin). This group prevents the transformation of HMG-COA into mevalonate. The statin group contains simvastatin, pravastatin, atorvastatin, lovastatin, pitavastatin, and rosuvastatin. This medicine group is classified according to the biopharmaceutics classification system (BCS) as a class II drug characterized by low solubility and high permeability. Thus, it causes low bioavailability in this group. In addition, it shows poor acid stability and is highly affected by the first-pass effect. Thus, rosuvastatin exhibits a low bioavailability of about 20% [3].

Rosuvastatin calcium (RSV) is a synthetic lipid-lowering agent, chemically known as (3R,5S,6E)-7-[4-(4-fluorophenyl)-6-isopropyl-2-[methyl(methylsulfonyl)amino]pyrimidin-5-yl])-3,5-dihydroxyhept-6-enoic acid hemicalcium salt [6]. Rosuvastatin, among other statins, is called a “super-statin,” causing a greater reduction in LDL than other statins of the same strength [7,8,9]. Several recent approaches have been published to improve rosuvastatin’s bioavailability using different mechanisms. Elsayed and his coworkers prepared forming nanoparticles in situ with the aid of Tween 80 and cetyl alcohol and filled in delayed-release capsules that improved the dissolution rate and bioavailability [10]. In addition, reducing the particle size of rosuvastatin using a wet milling technique by adding PVP 10% as a stabilizer enhanced its dissolution behavior to release 72% after 1 h [7]. Furthermore, the development of pullulan-based tablets containing flexible chitosomes of rosuvastatin calcium improved relative bioavailability by 30% to 36% compared to marketed drugs and pure rosuvastatin tablets [3]. Also, using caffeine and Soluplus^®^ to develop hydrotropic and micellar solubilization is another approach to directly compress rosuvastatin with improved bioavailability [11]. In addition, incorporating RSV into carboxylate cross-linked cyclodextrins improved its bioavailability [12]. Recently, González and his coworkers improved the bioavailability of RSV via its conversion to an amorphous form with a specific excipient to accelerate its dissolution onset by more than 90% in 10 min [13]. Also, RSV was incorporated with glimepiride in 3D-printed polypills formulated in a curcuma oil-based self-nanoemulsifying drug delivery system to treat patients with dyslipidemia and metabolic syndrome [14]. In addition, trials to formulate RSV as orodispersable films were performed with sophisticated and multi-stage procedures. The films produced by this work were evaluated for pharmacokinetic parameters, not for anti-dyslipidemic activity, as declared by our study [15].

Among other routes of administration, the oral route proved to yield optimum patient acceptability, as it is non-invasive and self-administered. There are many dosage forms administered orally. Some are wholly ingested; others can be chewed, dissolved in a specific solvent before taking, or adhered to the tongue or buccal cavity. Taking the dose via ingestion forced the active pharmaceutical ingredients into some challenges, like facing a low pH medium in the stomach, as many active pharmaceutical ingredients are unstable in acidic media. Also, some drugs are affected by first-pass metabolism prior to absorption. The relatively low bioavailability of some drugs after oral ingestion creates many challenges for developers to find a way to protect the drugs labile to these situations, like formulating them in delayed-release dosage forms.

Fast-dissolving films are considered a new oral dosage form that offers immediate action with a reasonable degree of protection from stomach acidity and the first-pass effect, as the dissolution and absorption phases are carried out in the oral cavity. It is a waterless dosage form and provides the action with a fast beginning. Fast-dissolving films, among other dosage forms, are highly accepted by pediatric and elderly patients due to their ease of use. Recently, many researchers published new polymer-based fast-dissolving films, e.g., fluoxetine [16], metoclopramide [17], lamotrigine [18], ondansetron hydrochloride [19], olanzapine [20], and tenoxicam [21]. Fast-dissolving films can be prepared using various techniques, such as solvent casting, characterized by combining the polymer solution with the plasticizer and drug solution, mixing, degassing, pouring into a suitable dish, and heating to evaporate the solvent [21]. Other methods include hot melt extrusion, semisolid casting, solid dispersion extrusion, and rolling [22]. A new approach was recently applied for preparing fast-dissolving dosage forms using a spinning agent that freely dissolves the drug of interest and is mixed with the polymeric solution [23].

Therefore, this work aimed to develop rosuvastatin calcium as a fast-dissolving film to be rapidly dissolved and absorbed in the buccal cavity. This approach helped to avoid the first-pass effect, protect the drug from degradation by stomach acidity, and subsequently improve the bioavailability of RSV.

## 2. Materials and Methods

### 2.1. Materials

Polyethylene glycol 400 (PEG 400) and rosuvastatin calcium were gifted from Egyptian International Pharmaceutical Industries Co. (10th of Ramadan, Egypt). Future Pharmaceutical Industries (Badr City, Egypt) provided hydroxypropyl methylcellulose (HPMC), viscosity 4000 cp, as a gift. Acetonitrile, ortho-phosphoric acid, methanol, and potassium dihydrogen phosphate were purchased from Merck (Darmstadt, Germany). Mannitol, Sorbitol, and Poloxamer 407 (P407, MW 40000) were obtained as a gift from Medical Union Pharmaceuticals (Ismailia, Egypt).

### 2.2. Methods

#### 2.2.1. Experimental Design

Firstly, many trials were carried out using a single-variable test to define the effective influential variables and their range to reach an optimum film with the required attributes. The formulation process was optimized using a 2^2+star^ central composite design, which studies two variables at three levels using ten runs. Table 1 lists the number of variables included in the design and their characteristics. Table 2 describes the central composite design’s layout. The effect of two factors, HPMC% (X_1_) and PEG 400% (X_2_), on the quality of the film was studied. A set of two center points per block and replicated center points were utilized to construct mathematical models and response surfaces using Statgraphics Centurion 18 software, Statgraphics Technologies, Inc. (Warrenton, VA, USA). After preparing and evaluating the prepared formulations, the data were statistically analyzed using one-way analysis of variance (ANOVA) to determine the significance of each variable for the *p*-value and F-ratio for each variable. The goal of the optimization was to minimize the disintegration time (Y_1_) and the thickness (Y_2_) and maximize the folding endurance (Y_3_) of the film.

#### 2.2.2. Preparation of RSV-FDFs

The films were prepared using the solvent-casting technique described before [22,24,25]. First, the required amount of HPMC (X_1_) was dispersed in 10 mL of water containing the sweetener and flavoring agent using a mechanical shaker (IKA, Staufen, Germany) for 6 h until completely dissolved. Conversely, RSV was dissolved in 5 mL of methanol solution in water (50% *v*/*v*). Then, both solutions were transferred into a beaker centered on a magnetic stirrer (IKA, Germany) and stirred for 30 min after adding the plasticizer (X_2_) and filling the volume to 25 mL. Then, the mixture was transferred to an ultrasonic bath for 15 min to degas, poured into a suitable glass dish, preserved in a refrigerator for 12 h to complete the swelling of the polymer, and then placed in an oven for 2 h at 40 °C for drying. The dried films were cut into strips, each of which contained 10 mg RSV.

#### 2.2.3. Characterization of RSV-FDFs

##### Physical Appearance

The physical appearance of the prepared RSV-FDFs was inspected for transparency, air bubbles, and color uniformity [21].

##### Content Uniformity, Average Weight, and Thickness

The average weight of three units was determined using a semi-micro analytical balance (Sartorius, Göttingen, Germany). Then, the thickness of these films was determined using a vernier caliber in three different places for each film. The average and the standard deviation were calculated and recorded [26].

Three units were selected at random from each formulation and dissolved in 100 mL of 20 mM phosphate buffer of pH 6.8. The obtained solution was measured spectrophotometrically at 242 nm using a UV instrument (UV1900, Shimadzu, Kyoto, Japan) [27].

##### Folding Endurance, Tensile Strength, and Elongation Percentage

The tensile strength, elongation percentage, and folding endurance were used to examine the film’s flexibility and durability. The folding endurance was determined by folding the film at an angle of 180° at one point until the film was deformed [28,29,30].

Using a laboratory-made instrument fabricated with two clamps, one of which was stabilized and fixed and the other freely moveable. A set of 10 gram weights was attached successively to the moveable part until the cracking or breaking of the film was examined. The tensile strength is the force applied to break the film by the Newton unit (N) over the cross-sectional area in square centimeters (cm^2^), as depicted in Equation (1).

The elongation percentage is determined by dividing the increment in length by the original length and then multiplying by 100, as described in Equation (2) [21,31].

(1) $$Tensile\;strength=\frac{Force\;applied\;(N)}{Cross\;sectional\;area\;(cm^{2})}$$

(2) $$Elongation\;percentage=\frac{Increment\;in\;length}{Original\;lenght}\times 100$$

##### Surface pH

The film pH was determined by placing the film in a suitable dish and wetting it with distilled water, then measuring the pH by immersing the electrode of the calibrated pH meter into the surface of the wetted film (Schott lab 850, Mainz, Germany) [32].

##### In Vitro Disintegration

The prepared films were tested for their in vitro disintegration by two methods. The first was adding a film strip to the disintegration tester (Copley Scientific Limited, Nottingham, UK) containing 900 mL of deionized Milli Q water (Millipore, Molsheim, France) at 37 °C. Then, the time for complete film disintegration was determined in triplicate [20]. The second was using the Petri dish method by adding a film strip to a Petri dish containing 3 mL of simulated salivary fluid (SSF) of pH 6.8 and applying gentle stirring to mimic the oral cavity condition; the time to disintegration was calculated with a stopwatch [33].

##### In Vitro Dissolution

The dissolution of RSV from the prepared films was determined by placing 3 units each of 10 mg from all formulas into a rotating basket (Apparatus I) rotated at 50 rpm in a 100 mL simulated salivary solution of pH 6.8. The dissolution tester (Logan, UT, USA) was maintained at 37 °C, and 5 mL samples were withdrawn after 2 min and suitably diluted before analysis to determine the RSV content using a UV spectrophotometer (Schimadzu, Japan) at 242 nm.

#### 2.2.4. In Vivo Pharmacokinetics Evaluation on Male Wistar Rats

##### Study Design

A one-period, open-label, single-dose, randomized, parallel design was implemented in the study. Two groups of male Wistar rats (6 rats per group) were administered a single dose of 20 mg/kg of the optimized RSV–FDF (test). At the same time, the marketed Crestor^®^ tablets (reference) (AstraZeneca, Cairo, Egypt) were administered in the same dose orally with water. The study was carried out at the International Center for Bioavailability, Pharmaceutical, and Clinical Research (ICBR, Cairo, Egypt). The Institutional Review Board/Independent Ethics Committee (IRB/IEC) at the ICBR formally reviewed the proposed study’s objective, design, conduct, and analysis. It approved the study protocol on 25 June 2022 with Ethical Approval Code RESH-0026.

##### Animal Handling and Blood Sampling

The animals were maintained in a controlled temperature with half-day morning and half-day night with access to food and water. At the time of administration, both formulations (RSV-FDF and oral tablets) were dissolved in 1% carboxymethyl cellulose. The corrected dose for each rat was administered orally using a gastric tube. Then, blood samples were collected in the following intervals: 0.5, 1, 2, 4, 6, 8, 12, 24, 36, 48, and 72 h. After each withdrawal interval, the samples were centrifugated for 10 min at 6000 RPM using a calibrated centrifuge (Eppendorf, Hamburg, Germany) and then frozen at −80 °C (Thermo, Sindelfingen, Germany). After the experiment, the samples were analyzed by the protein precipitation method. After thawing the samples and preparing the calibration curve from 25–3000 ng/mL, the samples, calibration, and quality control samples (QCs) were precipitated using acetonitrile (1:1) and then the samples were vortexed for 20 min at 5000 RPM. The resulting supernatant was transferred to high-performance liquid chromatography (HPLC) vial inserts.

##### Chromatographic Conditions

A volume of 50 µL was injected into the chromatographic system conditioned by a gradient elution of 0.1% phosphoric acid and acetonitrile with a Waters C18 stationary-phase Xbridge 250 × 4.6 mm with a 5 μm particle size. The R^2^ of the calibration line could not be less than 0.99, and the QC samples recovery needed to lie between 85 and 115%. The lower limit of quantitation was 25 ng/mL, and three quality control levels were determined in the following concentrations: 100, 1000, and 2000 ng/mL for QCL, QCM, and QCH, respectively. Then, the linearity equation was applied to determine the sample concentration after injecting the samples into a high-performance liquid chromatography apparatus (Waters, Milford, MA, USA) equipped with a PDA detector (Waters, Milford, MA, USA) maintained at 242 nm using Empower 3 software.

##### Pharmacokinetics Data Analysis

With the aid of the pharmacokinetics add-in PKsolver 2.0 software, the following parameters were measured and utilized to estimate the extravascular non-compartmental pharmacokinetics model: the time point of maximum drug concentration (T_max_), the highest concentration of RSV (C_max_), and the AUC, which is the area under the plasma concentration-time curve for each of the following: AUC_0–t_: from the 0–time point to the last measurable concentration using the trapezoidal method and AUC_0–∞_: the area under the concentration-time curve from the 0–time point to infinity. This was calculated via summation of the ratio of the last concentration in the plasma over the elimination rate constant with AUC_0–∞_, the area under the moment curve from zero, to the final AUMC, as well as the mean residence time (MRT). This was calculated by plotting the AUMC over the AUC and the total body clearance (Cl), which is calculated by plotting the dose per AUC. Also, the T half elimination (T_1/2_) was determined, which is calculated by dividing 0.693 by the K_el_, the elimination rate constant (K_el_). The apparent volume of distribution after non-intravenous administration at the terminal phase (Vd) was achieved by multiplying the total body clearance by the MRT. The relative bioavailability for the RSV-FDF versus the commercial tablets was calculated by dividing the AUC of the RSV-FDF by the AUC of the market tablets ×100.

#### 2.2.5. In Vivo Anti-Dyslipidemic Activity

The anti-dyslipidemic activity of the optimized RSV-FDF was compared with the marketed Crestor^®^ tablets. Male Wistar rats were used after injection with Poloxamer 407 to induce dyslipidemia 24 h before the experiment. Then, the rats were divided into three groups (3 rats per group). The optimized RSV-FDF was administered to the first group; marketed tablets were administrated to the second and third groups with no treatment as a model dyslipidemic group. Blood samples were taken at 0, 2, 6, 12, and 24 h and allowed to settle, and the serum was collected and analyzed to determine the lipid parameters (triglycerides, total cholesterol, LDLs, VLDLs, and HDLs). The in vitro diagnostic kits were used with the enzymatic colorimetric method for evaluation (Abcam Colorimetric/Fluorometric, ab65390, Waltham, Boston, USA).

#### 2.2.6. Statistical Analysis

The data for the pharmacokinetic and anti-dyslipidemic activities were statistically analyzed using GraphPad Prism 8 (GraphPad Software, Inc., La Jolla, CA, USA) as mean ± SD. Two-way analysis of variance (ANOVA) followed by Tukey’s multiple comparisons test was used to identify the significant difference between the studied groups. A *p*-value of less than 0.05 was statistically significant. The statistical significance between the pharmacokinetic parameters was determined using the Holm–Sidak method.

## 3. Results and Discussion

In the current work, optimized RSV-FDFs were developed by tailoring a polymeric matrix with the aid of hydroxypropyl methylcellulose and the plasticizing effect of glycerin. The formulation factors were investigated to determine their effects on the quality of the prepared FDFs and predict the optimum levels that produce the optimized formulation with the desired quality attributes. This optimized RSV-FDF was evaluated for its pharmacokinetic behavior and anti-dyslipidemic activity.

### 3.1. Formulation and Evaluation of RSV-FDFs

The evaluation of the prepared films’ physicomechanical properties is provided in Table 3. The films were found to be soft, clear, thin, and colorless, with no bubbles entrapped, and there were no issues during removal from the dish or the cutting procedures. The film clarity demonstrates that the drug was already soluble in the film polymer and thus supports the results of in vitro dissolution, which demonstrated immediate release after the disintegration of the film.

To assess the uniformity of the RSV distribution within the formula, five different places in each formula were analyzed to determine the drug content in each formula, and the results show that the drug was distributed uniformly throughout the films and within the accepted and required compendial specifications, with an RSD% of less than 10%. Also, the uniformity of weight in all films yielded an acceptable RSD%.

As the normal pH range of saliva lies between 6.2 and 7.6 [21], any acidic or basic pH distortion from normal salivary pH will cause irritation and patient noncompliance with the treatment protocol. All prepared films revealed a pH range of 6.5–6.62, ensuring no irritation to the oral cavity upon administration.

#### 3.1.1. Tensile Strength and Elongation Percentage

Table 3 shows no variability in the tensile strength (1.765–1.872 N/cm^3^) of the prepared films and no breakage of any of the films during the test, confirming the satisfactory mechanical property of the films.

Elongation percentage is typically a useful tool for describing the mechanical characteristics of film. Soft films are those that have low elongation percentages and tensile strengths. A soft and tough film has high tensile strength and high elongation, whereas a hard and brittle film has moderate tensile strength and low elongation [34]. In order to increase the elasticity and decrease the brittleness of the film, it is crucial to employ the right amount of plasticizer. According to the data in Table 3, the elongation percentage for F-1 and F-6 ranged from 10 to 88%, respectively. The HPMC percentage in the film strongly impacted this result. Also, the film’s PEG 400% was the most important factor, positively increasing the elongation percentage. The increased viscosity and brittleness of the manufactured films calls for more plasticizer use [35]. The plasticizer’s positive impact on elasticity can be explained by how it works to weaken the forces that hold polymer chains together, interrupt polymer chains, increase chain mobility, and improve the flexibility of the polymeric matrix, softening and extending the film matrix as previously reported [36,37,38].

#### 3.1.2. In Vitro Dissolution

The release of rosuvastatin from the films was rapid and precise, and all formulations showed complete dissolution within the first two minutes, as shown in Table 3. Thus, this may indicate the enhanced water solubility of the drug via dispersion with the polymer. Along with the use of water-soluble inert fillers that were reported to be used to form a highly water-soluble dispersion with active ingredients, the same also appeared with Choi et al., who correlated the solubility improvement of poorly soluble rivaroxaban to the dispersion of the drug in the polymeric solution [39].

### 3.2. Optimization of RSV-FDFs

Ten experimental runs were suggested by a 2^2+star^ central composite design to demonstrate the effect of the following independent variables: polymer percentage (X_1_) from 1 to 3% and plasticizer percentage (X_2_) from 1 to 2% on the disintegration time (Y_1_), and thickness (Y_2_) and folding endurance (Y_3_) of the prepared films.

#### 3.2.1. Estimation of the Quantitative Effects

Table 4 shows the statistical analysis of variance (ANOVA) of the Y_1_–Y_3_ response results. The factor effects of the model, F-ratio, and associated *p*-values for the responses are presented. A positive sign of the estimate indicates a synergistic effect, whereas a negative sign represents an antagonistic effect of the factor on the selected response. The table shows that X_1_ and X_2_ significantly synergistically affected all Y_1_–Y_3_ responses with *p*-values of less than 0.05.

**Figure 1:**
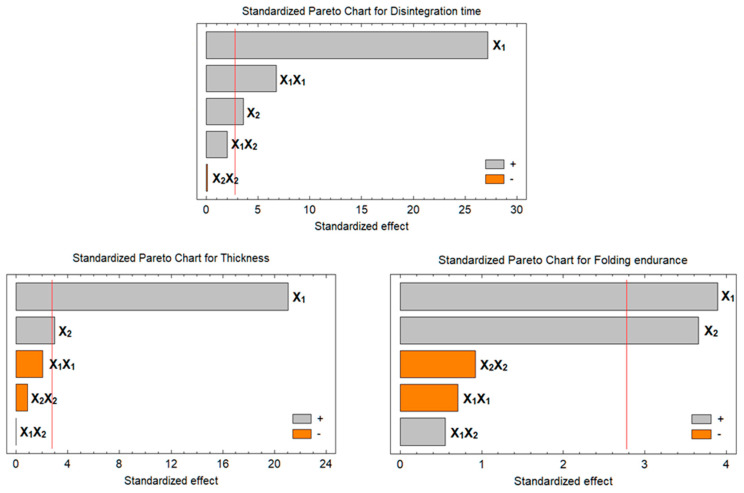
Pareto charts showing the effects of different variables on the responses of Y_1_–Y_3_. All the factors exceed the red line has a significant effect on the studied response.

**Figure 2:**
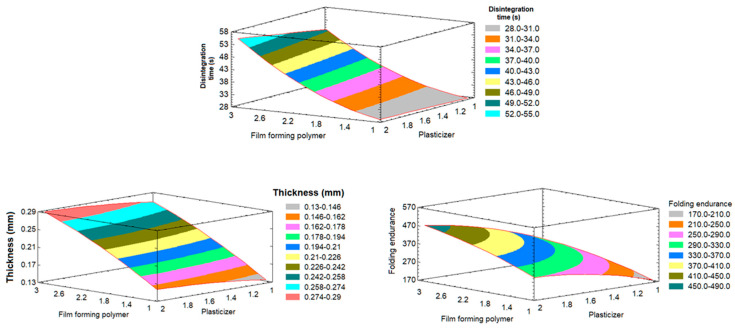
Effect of different variables (Y_1_–Y_3_) on the responses represented in the response surface plots.

The contour plots (Figure 1) demonstrate how several independent variables affected the responses of Y_1_–Y_3_. The response surface plots (Figure 2) made comparing each factor’s impact at a specific location in the design space easier. Plotting the response involved varying just one element over its range while keeping the other variables constant. The plot was plotted by Statgraphics^®^ 18 Centurion Software (Warrenton, VA, USA). These figures supplied information relating to the major contribution and influence of the factors on the responses. Figure 1 and Figure 2 declare that the effect of the polymer and plasticizer had a major effect on the prepared films.

##### Effect on the In Vitro Disintegration (Y_1_):

Regarding Y_1_, all formulas yielded a response of < 60 s except for F6, which disintegrated after 62 s; this may have been because of its higher content of HPMC, as reported by Rédai et al. [16]. The generated polynomial equation for Y_1_ is presented in Equation (3).

In vitro disintegration time (Y_1_) = 30.46 − 7.51 X_1_ − 1.09 X_2_ + 3.94 X_1_^2^ + 2.5 X_1_ X_2_ − 0.25 X_2_^2^ (3)

The dependent variable responded positively when the concentration of the independent variables X_1_ and X_2_ increased, as seen in Figure 1 and Figure 2.

The fast disintegration of the films at a low HPMC content (1%) could be explained by the fact that when polymeric content increased, the viscosity of the film increased, resulting in prolonged disintegration time [18,40].

The concentration of plasticizer (X_2_) had an impact on the fast-dissolving films’ in vitro disintegration as well, and there was a clear correlation as the plasticizer concentration increased. This behavior could be attributed to the increase in viscosity [21].

##### Effect on the Film Thickness

The average thickness of the films ranged from 0.11 to 0.31 mm. The film thickness, determined by a Vernier caliber, demonstrates the direct relationship between the percentage of polymer and the thickness of the film. When the polymer percentage increased, the thickness increased. The lowest polymer % in F1 had a thickness of 0.11 mm, and the highest polymer percentage in F6 had a thickness of 0.31 mm. These results were consistent with previously published data that related fast-dissolving film thickness to the percentage of polymer added [32,41]. The low polymeric content could explain the films’ thickness at a low HPMC content (1%), causing water to easily evaporate and leading to thin film [42].

The polynomial equation generated for Y_2_ is presented in Equation (4).

Film thickness (Y_2_) = −0.008 + 0.103 X_1_ + 0.06 X_2_ − 0.009 X_1_^2^ + 0.0 X_1×2_ − 0.015 X_2_^2^ (4)

##### Effect on the Folding Endurance

The durability of each formula was found to be related to the polymer and plasticizer content. The lowest amount of plasticizer, 0.79% in F10, displayed the lowest folding endurance of 155 times before breakage, whereas F8, containing 2.21% plasticizer, was folded up to 456 times with no breakage. Usually, good film can be folded 300 times or more [19]. The polynomial equation generated for Y_3_ is presented in Equation (5).

Folding endurance (Y_3_) = −205.91 + 102.83 X_1_ + 364.92 X_2_ − 18.13 X_1_^2^ + 30.0 X_1×2_ − 94.49 X_2_^2^ (5)

The response of Y_3_ ranged from 155 to 456 times for all formulations. Both X_1_ and X_2_ had significant model terms and showed positive responses due to increases in impart flexibility to the film, i.e., as the amount of polymer and plasticizer increased, the folding endurance also increased (Figure 1 and Figure 2).

The folding endurance of the films at a low content of HPMC (1%) could be related to the other effect of polymer on the thickness as the polymer decreases, yielding thin film that is more brittle compared to moderately thick film [31,43].

The content of plasticizer (X_2_) had an impact on the folding endurance of fast-dissolving films (FDF), and there was a direct correlation as plasticizer concentration increased. This behavior could be attributed to the effect of plasticizer, which is added to increase film’s elasticity, as it works by adding more viscosity and elasticity to the film [43].

#### 3.2.2. Preparation and Evaluation of Optimized Formula

According to the statistical analysis for the prepared formulations, the optimized formula achieved the lowest thickness and disintegration time and the highest folding endurance, achieved with 1.15% polymer and 2.1% plasticizer. The optimized formula yielded 0.17 mm thickness, 30 s disintegration time, and 320 times sustainability versus folding. Also, it had a surface pH of 6.6 and was completely dissolved within 2 min.

### 3.3. In Vivo Pharmacokinetics Evaluation

**Figure 3:**
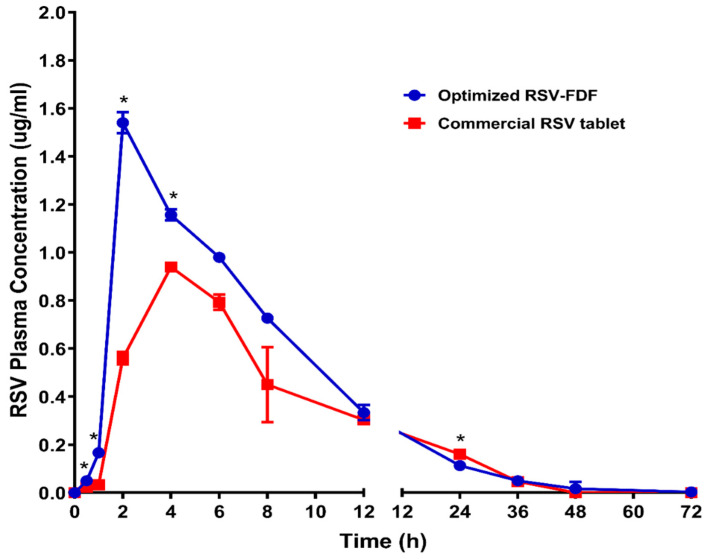
Rosuvastatin calcium plasma time concentration curves after administration of optimized RSV-FDFs and commercial RSV tablets. Data represent the mean value ± standard deviation (SD). * Significant at *p* < 0.05.

The plasma concentration-time curve acquired after dosing male Wistar rats with 20mg/kg rosuvastatin from the commercial product (M) and fast-dissolving film formula (F) is demonstrated in Figure 3. The pharmacokinetic parameters were calculated using WinNonLin^®^ 8.2 software (Princeton, NJ, USA) and are listed in Table 5. The absorption was monitored for a period of 72 h. The differences in T_max_ were used to evaluate these data. The RSV-FDF formula showed faster release, which was revealed in the reduction of the T_max_, and the extent of the absorbed drug improved, which appeared as a higher C_max_ for the RSV-FDF (1.540 ± 0.044 µg/mL) and as 0.940 ± 0.017 µg/mL for the marketed product. In comparison to the commercial formula, relative bioavailability improved by 32.5%.

Furthermore, the multiple *t*-test using the Holm–Sidak method revealed that C_max_, AUC_0–t_, AUC_0–inf_, and the clearance (Cl) showed significant differences between the optimized RSV-FDF and the commercial oral tablet, with *p*-values of 0.000025, 0.002059, 0.001063, and 0.002239, respectively.

The improvement in T_max_ directly referred to the origin of the formula of the RSV-FDF containing the rosuvastatin in the dissolved state, so there was only 1 min for average disintegration with no dissolution time required. At the same time, the marketed product needed more time for the disintegration and dissolution stages. The improvement in the RSV-FDF is also referred to as bypassing the first pass effect and protecting rosuvastatin from degradation in acidic media.

### 3.4. In Vivo Pharmacodynamics Evaluation

The hypolipidemic activity of rosuvastatin was referenced to prevent the synthesis of mevalonic acid from its precursor HMG-COA by inhibiting the enzyme HMG COA reductase, which decreases the lipid profile.

**Figure 4:**
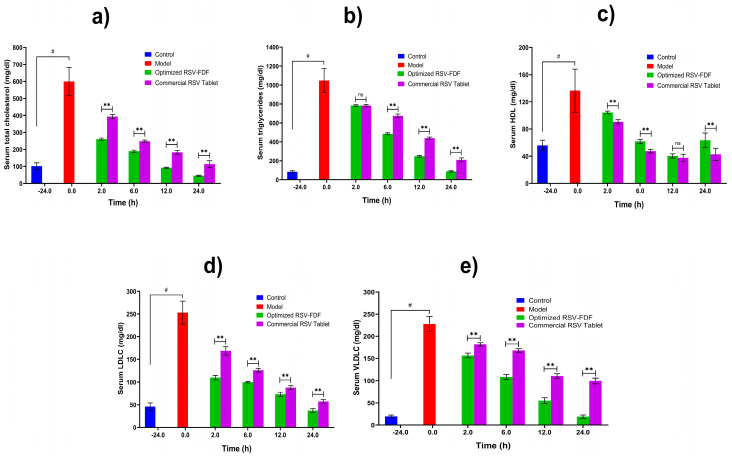
Lipid profiles of induced hyperlipidemic rats after single-dose administration of optimized RSV-FDFs and commercial RSV tablets. Data are presented as mean ± SD (n = 3). Note: # denotes a significant difference between normal and model at *p* < 0.05, ** denotes a significant difference between the optimized RSV-FDF and the commercial RSV tablets at *p* < 0.01, and ns denotes a non-significant difference. (**a**) the total serum cholesterol level in mg/dl, (**b**) the total serum triglyceride level mg/dl, (**c**) the serum HDL level mg/dl, (**d**) the serum LDL level mg/dl, and (**e**) the serum VLDL level mg/dl.

To study the efficiency of the RSV-FDF on the lipid profile (triglycerides, total cholesterol, LDLs, VLDLs, and HDLs) in rats with induced hyperlipidemia, Poloxamer 407 was administered to male Wistar rats 24 h prior to the experiment by to induce hyperlipidemia; then, the rats categorized into three groups (n = 3): negative control (C), commercial product (M), and RSV-FDF (O). Then, zero time samples were collected from each group to define the baseline for each parameter and the efficacy was determined for the O group, which showed a decrease in total cholesterol of 68.1% after 6 h from the zero time point, which is a significant difference in comparison to the M group, as the total cholesterol was reduced by 58.2% and the reduction In total cholesterol persisted for 24 h. The level of triglycerides also decreased by 56.4% for the O group, whereas the M group’s level of triglycerides was reduced by 37.6%. Also, for the LDLs, the O group showed better performance, as after 6 h, the level of LDLs in the O group decreased by about 60.6% compared to 50% in the marketed group. The following Figure 4 shows the graphical representation of the different parameters of the lipid profiles in the three groups.

## 4. Conclusions

The prepared fast-dissolving film formula containing rosuvastatin (RSV-FDF) yielded acceptable results regarding in vitro characterization and evaluation. The pharmacokinetics supported these findings, which revealed a significant improvement in relative bioavailability of 32.5%. The pharmacodynamic experiments also showed significant improvements for RSV-FDF compared to the commercial rosuvastatin tablets of a 50% reduction in triglyceride levels and a 21% reduction in LDL values. Therefore, the RSV-FDF can be considered a promising substitute for commercial tablets, although additional studies in humans and extra stability determinations should be carried out in the future.

## Figures and Tables

**Table 1:** Level of variables incorporated into the central composite design and their attributes.

**Independent Variables**	**Levels**		
	**Low**	**Medium**	**High**
X_1_ = film-forming polymer (HPMC) %	1	2	3
X_2_ = plasticizer (PEG 400) %	1	1.5	2
**Dependent Variables**	**Constraints**		
	**Low**	**High**	**Goal**
Y_1_ = disintegration time (s)	26	62	Minimize
Y_2_ = thickness (mm)	0.11	0.31	Minimize
Y_3_ = folding endurance	155	456	Maximize

**Table 2:** Layout of the experimental matrix of RSV-FDFs, with the independent and dependent variables proposed as suggested by the central composite design.

Run Code	Independent Variables		Dependent Variables		
	HPMC % (X_1_)	PEG 400 (X_2_)	Disintegration Time (Y_1_), s	Thickness (Y_2_), mm	Folding Endurance (Y_3_)
F1	0.59	1.5	26	0.11	189
F2	3.0	2.0	55	0.28	420
F3	3.0	1.0	49	0.27	320
F4	2.0	1.5	36	0.22	350
F5	2.0	1.5	37	0.23	354
F6	3.41	1.5	62	0.31	444
F7	1.0	1.0	29	0.14	229
F8	2.0	2.21	38	0.24	456
F9	1.0	2.0	30	0.15	269
F10	2.0	0.79	34	0.20	155

**Table 3:** Physical characterization of the prepared RSV-FDF batches.

Run Code	Surface pH	Average Weight (g)	Tensile Strength (N/cm^2^)	Percent Elongation (%)	Dissolution after 2 min (%)	RSV Content (%)
F1	6.5	0.02	1.765	10	98.61	103.69
F2	6.53	0.11	1.852	80	105.22	105.39
F3	6.6	0.10	1.843	64	105.56	103.88
F4	6.62	0.08	1.814	74	101.79	104.91
F5	6.6	0.08	1.816	72	105.69	105.16
F6	6.6	0.12	1.872	88	101.52	105.37
F7	6.58	0.03	1.758	16	98.68	104.72
F8	6.61	0.09	1.828	76	100.33	105.33
F9	6.62	0.03	1.778	70	97.22	105.55
F10	6.58	0.07	1.807	20	102.65	104.97

Note: mean ± SD used to present the data (n = 3).

**Table 4:** Statistical analysis of variance (ANOVA) of the Y_1_–Y_3_ response results.

Factors	Disintegration Time (Y_1_), s			Thickness (Y_2_), mm			Folding Endurance (Y_3_)		
	Estimate	F-Ratio	*p*-Value	Estimate	F-Ratio	*p*-Value	Estimate	F-Ratio	*p*-Value
X_1_	23.98	739.58	0.0001 *	0.14	443.28	0.0001 *	150.66	15.17	0.0176 *
X_2_	3.16	12.88	0.0230 *	0.02	8.82	0.0412 *	141.42	13.36	0.0217 *
X_1_X_1_	7.88	45.59	0.0025 *	−0.02	4.21	0.1094	−36.25	0.50	0.5178
X_1_X_2_	2.50	4.02	0.1155	0.00	0.00	1.0000	30.00	0.30	0.6126
X_2_X_2_	−0.13	0.01	0.9198	−0.01	0.77	0.4288	−47.25	0.85	0.4081
R^2^	99.51			99.13			88.17		
Adj. R^2^	98.89			98.04			73.37		
SEE	1.25			0.009			54.71		
MAE	0.68			0.005			28.98		

Note: * Significant effect of factors on individual responses (*p*-value less than 0.05). Abbreviations: X_1_, film-forming polymer (HPMC) %; X_2_, plasticizer (PEG 400) %; X_1_X_2_, the concept describing how the factors interact; X_1_X_1_, and X_2_X_2_ are the quadratic terms between the factors; R^2^, R-squared; Adj-R^2^, adjusted R-squared; SEE, standard error of estimate; and MAE, mean absolute error.

**Table 5:** Pharmacokinetic parameters of the optimized RSV-FDF versus the marketed RSV tablet after administration of 20 mg/kg in rats orally (n = 6, data expressed as average ± SD).

Parameter	Unit	Optimized RSV-FDFs		Marketed RSV Tablets	
		Average	STDEV	Average	STDEV
Lambda_z	1/h	0.072	0.020	0.084	0.004
t_1/2_	h	10.172	2.716	8.231	0.417
T_max_	h	2.000	0.000	4.000	0.000
C_max_	µg/mL	1.540 *	0.044	0.940	0.017
AUC_0–t_	µg/mL.h	13.680 *	0.622	10.320	0.531
AUC_0–inf_	µg/mL.h	14.178 *	0.331	10.874	0.589
AUMC_0–inf_	µg/mL.h^2^	166.681	27.408	141.365	6.273
MRT_0–inf_	h	11.731	1.656	13.005	0.188
Vz	(mg)/(µg/mL)	20.629	5.057	21.894	1.803
Cl	(mg)/(µg/mL)/h	1.411 *	0.033	1.843	0.102

Note: * denotes a significant difference between values of the optimized RSV-FDF and values of the marketed RSV tablet at *p* < 0.05.

## Data Availability

Data are contained within the article.
